# High-Frequency Intermuscular Coherence between Arm Muscles during Robot-Mediated Motor Adaptation

**DOI:** 10.3389/fphys.2016.00668

**Published:** 2017-01-09

**Authors:** Sara Pizzamiglio, Martina De Lillo, Usman Naeem, Hassan Abdalla, Duncan L. Turner

**Affiliations:** ^1^Neuroplasticity and Neurorehabilitation Doctoral Training Programme, Neurorehabilitation Unit, School of Health, Sport and Bioscience, University of East LondonLondon, UK; ^2^Department of Computer Science, School of Architecture, Computing and Engineering, University of East LondonLondon, UK; ^3^University College London Partners Centre for NeurorehabilitationLondon, UK

**Keywords:** force-field, motor adaptation, neuromuscular control, muscle co-contraction, intermuscular coherence

## Abstract

Adaptation of arm reaching in a novel force field involves co-contraction of upper limb muscles, but it is not known how the co-ordination of multiple muscle activation is orchestrated. We have used intermuscular coherence (IMC) to test whether a coherent intermuscular coupling between muscle pairs is responsible for novel patterns of activation during adaptation of reaching in a force field. Subjects (*N* = 16) performed reaching trials during a null force field, then during a velocity-dependent force field and then again during a null force field. Reaching trajectory error increased during early adaptation to the force-field and subsequently decreased during later adaptation. Co-contraction in the majority of all possible muscle pairs also increased during early adaptation and decreased during later adaptation. In contrast, IMC increased during later adaptation and only in a subset of muscle pairs. IMC consistently occurred in frequencies between ~40–100 Hz and during the period of arm movement, suggesting that a coherent intermuscular coupling between those muscles contributing to adaptation enable a reduction in wasteful co-contraction and energetic cost during reaching.

## Introduction

Motor adaptation to a physical disturbance is a dynamic process and involves a complex interaction of central (neural) and peripheral (muscular) systems (Lemon, 2008; Rosenbaum, 2009; Gandolla et al., 2014). Conceptually, the underlying mechanism operating during motor adaptation has been viewed as the combination of online error correction, as a result of sensory (mainly proprioceptive) feedback and the development of a new internal model of the new skill (Shadmehr and Brashers-Krug, 1997; Wolpert and Ghahramani, 2000; Krakauer and Shadmehr, 2006).

Motor adaptation of arm reaching to force field perturbations involves changes in the activation pattern of upper limb muscles that may serve to reduce online errors and may represent the development of a new internal model (Osu et al., 2003; Milner and Franklin, 2005; Orban de Xivry et al., 2013). To date however, muscle activation patterns have been studied using relatively straightforward approaches such as integrated muscle activation during certain time periods (Thoroughman and Shadmehr, 1999; Milner and Franklin, 2005). Indices such as co-contraction ratios have also been computed to give an estimate of energy-expensive wasted co-contraction (Darainy and Ostry, 2008; Huang and Ahmed, 2014) or put forward as a mechanism for stiffening the arm during motor adaptation (Milner et al., 1995; Koshland et al., 2000). Whilst these techniques can describe a “muscle output” signal, they may only indirectly infer that there are changes in input neural signals during motor adaptation. As an alternative approach, we have used intermuscular coherence (IMC) to identify possible neural mechanisms contributing to the optimization of behavioral performance during motor adaptation (Halliday et al., 1995; Halliday and Rosenberg, 2000). IMC has been used for the evaluation of coherent activation in muscle pairs during isometric contraction tasks (Baker et al., 1999; Kilner et al., 1999; Poston et al., 2010; Semmler et al., 2013; Jesunathadas et al., 2013), tremor (Halliday et al., 2000; van Rootselaar et al., 2006; van der Stouwe et al., 2015), and more recently in rhythmic movement such as pedaling (De Marchis et al., 2015) and stepping (Chang et al., 2012). Changes in different frequency bands of coherence may confer information on the changes in descending neural signals (grip task, IMC in 0–35 Hz range, Danna-Dos Santos et al., 2010; precision grip and ankle dorsiflexion task, IMC in 15–30 Hz range Fisher et al., 2012; precision grip tasks during sustained extension/flexion of elbow joint, IMC in 13–25 Hz range, Lee et al., 2014), on the status of functional recovery of neural structures after injury (force-tracking precision grip task, IMC in 30–46 Hz range, Nishimura et al., 2009) or of impaired motor skills (reaching, IMC in 0–11 Hz range, Kisiel-Sajewicz et al., 2011). IMC has been also recently shown to increase between muscles pairs that are more strongly coordinated during specific motor tasks (bimanual coordination, de Vries et al., 2016; upper-limb isometric contractions to control a myoelectric cursor, Nazarpour et al., 2012), experimentally supporting the hypothesis that multiple muscles coordination may be the result of a neural synchronization strategy of cortical origins (Farmer, 1998). Here we use IMC to test the hypothesis that there is an increase in coherent muscle activation during motor adaptation and that it is related to formation of a new behavioral optimization strategy reducing reaching errors as well as co-contraction.

## Methods

### Ethical approval

Nineteen right-handed healthy adults [age mean (± std) = 28.2 (± 4.6), 3 male/16 female] with no neurological, neuromuscular, and/or orthopedic disease(s) history agreed to participate by giving written informed consent in this study which was approved by the University of East London Ethics Committee (UREC_1415_29). All experiments were conducted in accordance with the Declaration of Helsinki. Data of three subjects (1 male, 2 female) were discarded because of problems during data acquisition, leaving a total of sixteen subjects [age mean (± std) = 27.9 (± 4.8), 2 male/14 female]. No gender effect was found within the results.

### Reaching trials

Subjects sat in a comfortable chair, which was directly in front of a shoulder/arm manipulandum workstation (MIT-Manus, Interactive Motion Technologies, Cambridge, MA, USA). The subjects were then required to grasp the end-effector handle with their right hand (70° shoulder extension, 120° elbow flexion, semi-pronated arm). The subject's forearm was placed in a custom-made thermoplastic trough attached to the joystick for the support of the reaching arm against gravity. The shoulders were at the same level of the end-effector and safety belt straps were used to restrict trunk movements. A vertical display screen situated at eye-level provided online feedback on the position of the handle. Subjects were instructed to perform straight-line reaching trials (15 cm trajectory length) from a central starting point (1 cm diameter on screen) to a peripheral target (1 cm diameter on screen) within 1.0–1.2 s. The arm was repositioned to the central position by the robot after each voluntary reach trial (i.e., passive arm return so as not to interfere with the motor adaptation process).

### Motor adaptation protocol

The experimental protocol was based on 3 conditions, each composed of 96 reaching trials (total of 288 trials per experiment). The first condition (*Familiarization*) was performed in a null force-field and was intended to enable naive subjects to become familiar with the reaching task. During the second condition (*Motor Adaptation*), the robot applied a 25 Ns/m velocity-dependent force-field in the clockwise direction, perpendicular to the trajectory of the joystick. The third condition (*Wash Out*) was performed in a null force-field once again. The reaching task was always in one direction (135°; north-west direction on target board) in order to maximize the adaptation process and avoid complex patterns of muscle activity as a result of movement in plural directions. A clockwise force-field perturbation was employed in order to modify muscle activity in upper limb flexors (cf. Thoroughman and Shadmehr, 1999).

### Recording techniques

Kinematic parameters of each reach trial were recorded by the robotic device with sensors incorporated in the robot actuators. End-effector position and velocity (along the x and y axes), and exerted forces (along x, y, and z axes; N) were sampled at 200 Hz and stored for off-line analysis. Electromyographic activity (EMG; μV) was recorded from the right arm Anterior Deltoid (AD), Posterior Deltoid (PD), Biceps Brachii (BB), Triceps Brachii (TB), Extensor Carpi Radialis (ECR), Flexor Carpi Radialis (FCR), and Brachioradialis (BR) muscles. Bipolar superficial electrodes with a fixed 1.5 cm inter-electrode distance were positioned on each muscle according to a belly-belly montage, according to SENIAM guidelines (Hermens et al., 2000). Data were sampled at 1 kHz with a gain of 100 mV for the Biceps Brachii analog channel and 300 mV for all the other analog channels and were digitized via a 14 bit analog-to-digital convertor (DataLog EMG system, Biometrics Ltd, Newport, UK). In order to synchronize kinematic and physiological signals, the robotic device sent a TTL pulse at each visual cue (i.e., trigger at the beginning of a trial, time = 0 s) via a BNC cable to the EMG system.

### Data analysis

Offline data analyses were run in MatLab 2013b (The MathWorks, Inc.). Kinematic data from the robot were interpolated in order to match the sampling rate of the EMG signals. Reaching movements were described by a starting time point (movement onset defined by a speed profile exceeding 0.03 m s^−1^) and by an end time point (movement offset defined by a speed profile lower than 0.03 m s^−1^).

#### Kinematics and kinetics

Trial-by-trial trajectory error was quantified, using established methods, by calculating the summed error (m), defined as the absolute cumulative perpendicular distance (values are only positives regardless of path directionality) between the actual trajectory and the ideal straight line connecting the central starting point and the peripheral target. It consists of a measure of error for the whole duration of the reaching movement, from movement onset to movement offset and captures both changes in trajectory and movement duration that may occur during motor adaptation (Osu et al., 2003; Hunter et al., 2009). Additional measures included peak velocity (m s^−1^) and peak x-y planar force production by the subject (*N*) during the reach trial.

#### Basic muscle activity and co-contraction

Trial-by-trial raw EMG data were first de-trended, high-pass filtered at 45 Hz (Butterworth, order 3, dual-pass fashion to avoid phase-lag), notch filtered (50 Hz, IIR Comb Notching filter as designed in MatLab, order 20) and rectified. The high-pass filter choice for basic muscle activity and co-contraction analysis was based on the commonly accepted knowledge that EMG signals may be contaminated by intrinsic low-frequency noise sources (De Luca et al., 2010). The chosen cut-off frequency ensures that all the possible noise and movement artifacts are excluded from the signal. Each muscle activity was normalized to the maximum value registered in that muscle across the whole experimental recording (i.e., activation ratio, %) in order to minimize variability across subjects due to possible variation in electrode-skin impedances.

After preprocessing of the data, maximum EMG activation (Peak EMG; μV) and latency (Peak EMG latency relative to movement onset; ms) were firstly calculated for each trial within a time period ranging from movement onset and movement offset. Trial-by-trial filtered and rectified EMG signals were secondly used to assess muscle pair co-contraction between all the possible combinations of muscles of the right arm (i.e., 21 pairs in total). Analyses were conducted according to literature on “wasted contraction” (Thoroughman and Shadmehr, 1999; Gribble et al., 2003; Huang and Ahmed, 2014). Given normalized muscle activities of two muscles (i.e., one pair) during one trial, the minimum EMG activity level between the two profiles for each time point between the visual cue to reach and 3 s after was considered, creating a new co-contraction profile. Co-activity was evaluated for each subject and muscle pair in each trial.

#### Intermuscular coherence (IMC)

Coherence is a measure of oscillatory synchrony between signals in the frequency domain, computed by evaluating auto (S_xy_) and cross spectra (S_xx_, S_yy_) of L = 16 trials in a “pooled” fashion and then normalizing them according to Equation (1):

(1) $$C_{xy}(f)=\frac{|\sum_{i=1}^{L}S_{xy_{i}}(f){|}^{2}}{\left(\sum_{i=1}^{L}S_{xx_{i}}\right)\;\cdot \;\left(\sum_{i=1}^{L}S_{yy_{i}}\right)}$$

This step is performed every 16 trials in each condition and for each subject separately. Coherence reflects the consistency of the phase difference between the two sources at a given frequency. It is evaluated in specific frequency bins whose width is determined by the chosen frequency resolution and its values range from 0 to 1.

In order to assess IMC, raw EMG data were de-trended, band-pass filtered between 2 Hz and 100 Hz (Butterworth, order 3, dual-pass fashion to avoid phase-lag) and notch filtered (50 Hz, IIR Comb Notching filter as designed in MatLab, order 20). Offline filter settings are variable across different types of analysis: Here the band-pass filter choice for IMC analysis removes the low frequency modulation related to movement *per se* (~1 Hz). Blind Source Separation (BSS, Kilner et al., 2002) was also run across all the possible combinations of muscles of the right arm (i.e., 21 pairs in total) in order to minimize the muscle activity contamination in the recording in one muscle in each pair by the other. We used the Joint Approximate Diagonalization of Eigenmatrices (JADE) algorithm, which employs the fourth order statistics to automatically repress Gaussian background noises and enhance the non-Gaussian source signals (Cardoso and Souloumiac, 1993). This BSS algorithm does not require parameter tuning for good performance and has been recently showed to yield reliable results in neurophysiological studies (Correa et al., 2007) and for reducing crosstalk in EMG recordings (Léouffre et al., 2013; Chowdhury et al., 2013; Garcia and Keller, 2008). Time-frequency representations of coherence were computed for each pair of muscles in each trial over a period of 3 s from the visual cue to reach and for frequencies from 2 to 100 Hz. Cross- and auto-spectra were estimated with the Welch's periodogram method: Signals were divided into overlapping windows (Hamming window, 200 ms length and 75% overlap), then zero-padded to match the fast Fourier transform length of 1000 samples and eventually smoothed with a Gaussian window of 500 ms (Mehrkanoon et al., 2013). The result of this process was a 100 (frequency bins) × 60 (temporal bins) pixel matrix, where each “time-frequency spectrogram coherence sample” which we define here as a pixel, contained the coherence value associated to a given frequency at that specific time point. Each pixel thus had a frequency increment of 1 Hz and a temporal increment of 50 ms. The time-period-of-interest (3 s post-visual cue) was dictated by our interest in the temporal evolution of coherence over the reaching movement and during the period after the movement (i.e., the quasi-isometric holding-phase at the peripheral target before the robot passively returned the joystick to the start position after 3 s). A control analysis has been run defining trials around Movement Onset (−500 ms) before Onset to +2500 ms after Onset and results are reported in Supplementary Material; see Supplementary Figure 6 and Supplementary Table 3).

### Statistics

#### Kinematics, kinetics, basic muscle activity, and co-contraction

All measures of motor adaptation were assessed trial-by-trial for each subject and then averaged for 16 trials across each block (i.e., 6 blocks per condition) and across subjects (*N* = 16). Statistical analysis of motor adaptation measures then focused on differences between 8 blocks of major interest: Block 6 (*Familiarization trials* 81–96), block 7 (*Motor Adaptation* trials 1–16), block 8 (*Motor Adaptation* trials 17–32), block 9 (*Motor Adaptation* trials 33–48), block 10 (*Motor Adaptation* trials 49–64), block 11 (*Motor Adaptation* trials 65–80), block 12 (*Motor Adaptation* trials 81–96) and block 18 (*Wash Out* trials 81–96).

Statistical analyses were run through SPSS 20 (IBM) and MatLab 2013b. Averaged block data were first tested for normality with the Kolmogorov-Smirnoff test. The vast majority of data were normally distributed. Where data were not normally distributed, we also conducted non-parametric analyses, but in all circumstances these did not yield different results to parametric analyses so are not presented here. For each measure, 1 way repeated measures analysis of variance with factor “block” (repeated measures ANOVA; 8 blocks) was performed in order to highlight the presence of any variance across blocks. For each kinematic measure, peak force, basic muscle activity or co-contraction measure, paired-sample *T*-tests, with False Discovery Rate correction for multiple comparisons, were employed to define differences between block 6 (familiarization) and blocks 7–12 during adaptation and at the end of washout (block 18). Considering α = 0.05, paired-samples *T*-tests were considered statistically significant according to False Discovery Rate correction for multiple comparisons, with number of repeated test = 7, i.e., Block 6 vs. Block_i_ with i = 7, …, 12, 18.

#### Intermuscular coherence (IMC)

Given the dynamic force development in our task (*cf*. isometric tasks), we first evaluated the presence of synchronization between pairs of EMG signals through time-frequency pixelated maps of IMC. Previous work has used such a methodology for non-stationary electrophysiological data and confirmed the validity of the results as long as the two considered signals are independent (Zhan et al., 2006). In order to assess the basic patterns of time-frequency coherence (i.e., to observe if coherence values are significantly different from 0), for every subject and for every block specific time-frequency pixels in the IMC maps were considered statistically significant at *P* < α if they exceeded the confidence limit threshold, where L = 16 trials in this study (Halliday and Rosenberg, 1999):

(2) $$Thr=1-\alpha^{1/(L\;-\;1)}$$

Significant coherence time-frequency pixels were computed at 95% (α = 0.05) level of significance for each subject. Comparison of threshold values analysis is reported in Supplementary Material (see Supplementary Figures 3–5). Results of a typical subject and of the group average are presented as colored maps of the time-frequency coherence matrix in which each pixel is given a color code (dark blue = 0, bright red = 1, **Figure 4**).

Absolute peak IMC values were extracted from each block and for each subject from the time-frequency pixel region-of-interest and, along with their corresponding peak IMC frequency and peak IMC latency, were tested for normality with Kolmogorov-Smirnoff tests and subsequently treated by 1 way repeated measures ANOVA with False Discovery Rate corrected *post-hoc* paired *T*-tests as before. All values are reported as mean (standard deviation, *SD*). Additional correlation analyses between peak IMC, peak Co-contraction and Summed Error are reported in Supplementary Material (see Supplementary Table 1).

## Results

### Measures of motor adaptation

#### Kinematics and kinetics

**Figure 1:**
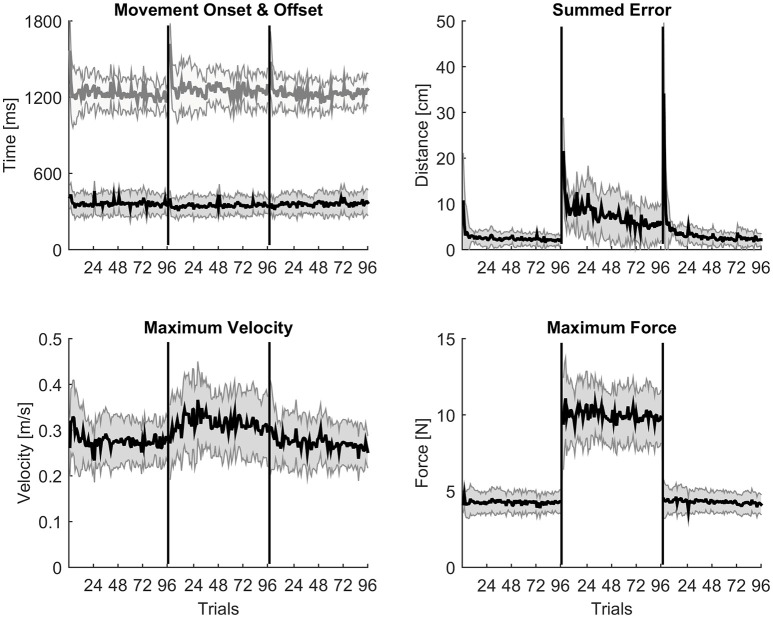
**Trial-by-trial kinematic evidences of motor adaptation**. A trial-by-trial population average (*N* = 16) profile with shaded standard deviation for each kinematic measure across the three experimental conditions. Movement onset and offset are almost constant throughout the whole experimental protocol; peak velocity and peak force show a typical constant increase of values during the adaptation condition; summed error instead slowly decreases trial by trial during adaptation as expected.

Each measure is represented by an averaged trial-by-trial trend (thick lines) and standard deviation (shaded areas, Figure 1), and block-by-block descriptive statistics across the three conditions are reported in Table 1. Repeated measures ANOVA values were significant for all the kinematic and kinetic measures (all *F* > 2.818; all *p* < 0.010). According to paired-samples *T*-tests, all motor adaptation blocks were significantly higher than block 6 for movement offset (all *p* < 0.007), summed error (all *p* < 0.001), peak velocity (all *p* < 0.007), and peak force (all *p* < 0.001). Movement onset only decreased in block 7 compared to block 6 (corrected paired *T*-test: *p* = 0.005). All measures returned to baseline after wash out illustrated by the comparison between block 6 and block 18.

**Table 1:** **Kinematics and electromyography results**.

	**Kinematics and electromyography results**								
	**Null field Block 6**	**Force field Block 7**	**Force field Block 8**	**Force field Block 9**	**Force field Block 10**	**Force field Block 11**	**Force field Block 12**	**Null field Block 18**	**Anova *p***
**KINEMATICS**									
Move onset (ms)	353 (67)	330 (54)	341 (65)	338 (68)	340 (57)	343 (61)	349 (56)	361 (77)	0.001
Move offset (ms)	1200 (45)	1279 (121)	1260 (70) ^*^	1261 (53) ^*^	1251 (63) ^*^	1252 (52) ^*^	1250 (47) ^*^	1233 (49)	0.010
Peak velocity (ms^−1^)	0.27 (0.03)	0.3 (0.06) ^*^	0.33 (0.07) ^*^	0.32 (0.06) ^*^	0.31 (0.06) ^*^	0.31 (0.06) ^*^	0.03 (0.06) ^*^	0.26 (0.04)	0.001
Peak force (*N*)	4.25 (0.5)	10.0 (1.8) ^*^	10.2 (1.7) ^*^	9.9 (1.6) ^*^	9.9 (1.7) ^*^	9.9 (1.7) ^*^	9.9 (1.6) ^*^	4.2 (0.6)	0.001
Summed error (m)	2.05 (0.8)	9.78 (2.4) ^*^	9.04 (4.0) ^*^	7.31 (3.9) ^*^	6.91 (4.4) ^*^	5.80 (3.8) ^*^	5.35 (3.3) ^*^	2.33 (1.2)	0.001
**EMG**									
**Peak (%)**									
AD	0.46 (0.21)	0.43 (0.18)	0.39 (0.18)	0.40 (0.19)	0.40 (0.19)	0.40 (0.19)	0.40 (0.19)	0.39 (0.18)	NS
PD	0.21 (0.16)	0.9 (0.19) ^*^	0.40 (0.17) ^*^	0.29 (0.17) ^*^	0.27 (0.18) ^*^	0.26 (0.17)	0.23 (0.18)	0.15 (0.13)	0.008
BB	0.23 (0.11)	0.51 (0.18) ^*^	0.47 (0.20) ^*^	0.45 (0.20) ^*^	0.44 (0.18) ^*^	0.44 (0.19) ^*^	0.46 (0.18) ^*^	0.20 (0.13)	0.001
TB	0.30 (0.17)	0.43 (0.15) ^*^	0.34 (0.15)	0.29 (0.15)	0.27 (0.14)	0.27 (0.14)	0.26 (0.14)	0.22 (0.13)	0.001
BR	0.17 (0.10)	0.46 (0.12) ^*^	0.38 (0.15) ^*^	0.36 (0.15) ^*^	0.36 (0.14) ^*^	0.33 (0.14) ^*^	0.35 (0.16) ^*^	0.12 (0.08)	0.001
FCR	0.18 (0.13)	0.55 (0.14) ^*^	0.51 (0.16) ^*^	0.46 (0.14) ^*^	0.43 (0.14) ^*^	0.42 (0.15) ^*^	0.42 (0.15) ^*^	0.16 (0.09)	0.001
ECR	0.16 (0.11)	0.41 (0.14) ^*^	0.33 (0.16) ^*^	0.27 (0.13) ^*^	0.26 (0.14) ^*^	0.24 (0.13) ^*^	0.22 (0.16)	0.16 (0.12)	0.001
**Peak Latency (ms)**									
AD	472 (150)	424 (168)	334 (130) ^*^	382 (168) ^*^	361 (179) ^*^	360 (131) ^*^	325 (153) ^*^	448 (172)	0.001
PD	448 (100)	641 (141) ^*^	591 (101) ^*^	626 (88) ^*^	555 (128)	566 (108) ^*^	551 (109)	486 (113)	0.001
BB	533 (138)	462 (172)	399 (124) ^*^	367 (148) ^*^	368 (125) ^*^	354 (124) ^*^	317 (99) ^*^	534 (129)	0.001
TB	532 (106)	625 (134)	577 (108)	601 (134)	568 (157)	575 (147)	523 (156)	606 (115)	0.023
BR	458 (141)	460 (174)	448 (136)	421 (150)	389 (149)	379 (150)	351 (144)	487 (99)	0.002
FCR	474 (148)	452 (135)	380 (141)	328 (141) ^*^	288 (92) ^*^	298 (86) ^*^	297 (103) ^*^	536 (127)	0.001
ECR	498 (101)	579 (161)	510 (104)	522 (122)	475 (121)	470 (118)	481 (127)	492 (141)	NS

Block-by-block (n = 8) mean (SD) of five kinematic parameters (movement onset, movement offset, peak velocity, peak force, and summed error) and two EMG measures (Peak EMG and Peak EMG latency relative to movement onset) for all muscles [Anterior Deltoid (AD), Posterior Deltoid (PD), Biceps Brachii (BB), Triceps Brachii (TB), Extensor Carpi Radialis (ECR), Flexor Carpi Radialis (FCR)]. Repeated measures ANOVA p-values are reported in the last column on the right, and statistically significant paired-samples t-test corrected for multiple comparisons (FDR) are highlighted with

* *(N = 16, Block 6 vs. Block_i_ with i = 7, …,12, 18; 7 comparisons)*.

#### Basic muscle activity

**Figure 2:**
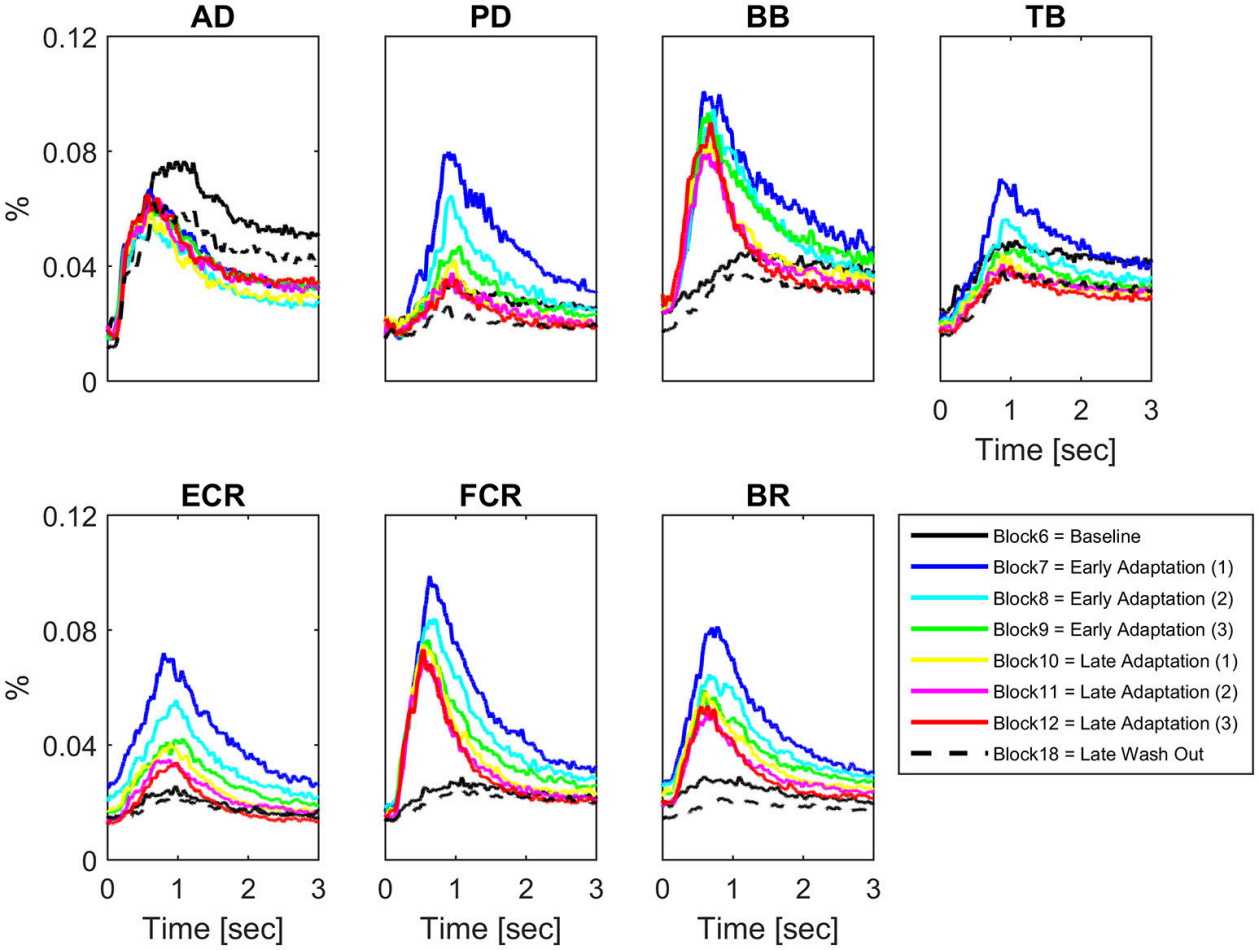
**Block-by-block muscle-specific activation profiles**. For each muscle block-by-block average (*N* = 16) activation profile (from visual cue to 3 s afterwards) have been color coded to describe the evolution of muscle-specific activation over the adaptation period. A clear pattern of adaptation to the clockwise force-field is present in Biceps Brachii and Flexor Carpi Radialis.

Some muscles demonstrated increases in peak EMG amplitude across conditions on a block-by-block basis; others had more complex patterns of activity change, whilst others did not change activity across conditions (Figure 2). Table 1 presents the results of the 1 way repeated measures ANOVA for peak EMG amplitude, whereby FCR (*F* = 31.8, *p* < 0.001), BR (*F* = 25.3, *p* < 0.001), PD (*F* = 24, *p* < 0.001), ECR (*F* = 20.7, *p* < 0.001), BB (*F* = 18.9, *p* < 0.001), and TB (*F* = 8.7, *p* < 0.001), were statistically different across blocks, but AD was not (*F* = 0.801, *p* > 0.05). Paired-sample *T*-tests demonstrated that BB, FCR, and BR peak EMG amplitude (*p* < 0.001) increased during early adaptation compared to baseline and did not show any further change during later adaptation. PD, TB, and ECR peak EMG amplitude increased in the early adaptation period with respect to baseline (all *p* < 0.008), but then progressively decreased toward baseline again by block 8 for TB, block 11 for PD, and block 12 for ECR. In some muscles, the increase of peak EMG amplitude during adaptation was accompanied by a shortening of peak EMG amplitude latency: This was the case for BB and FCR, whose peak EMG amplitude latency shortened in later adaptation (paired *T*-tests for the last four blocks, *p* < 0.002).

#### Muscle co-contraction

**Figure 3:**
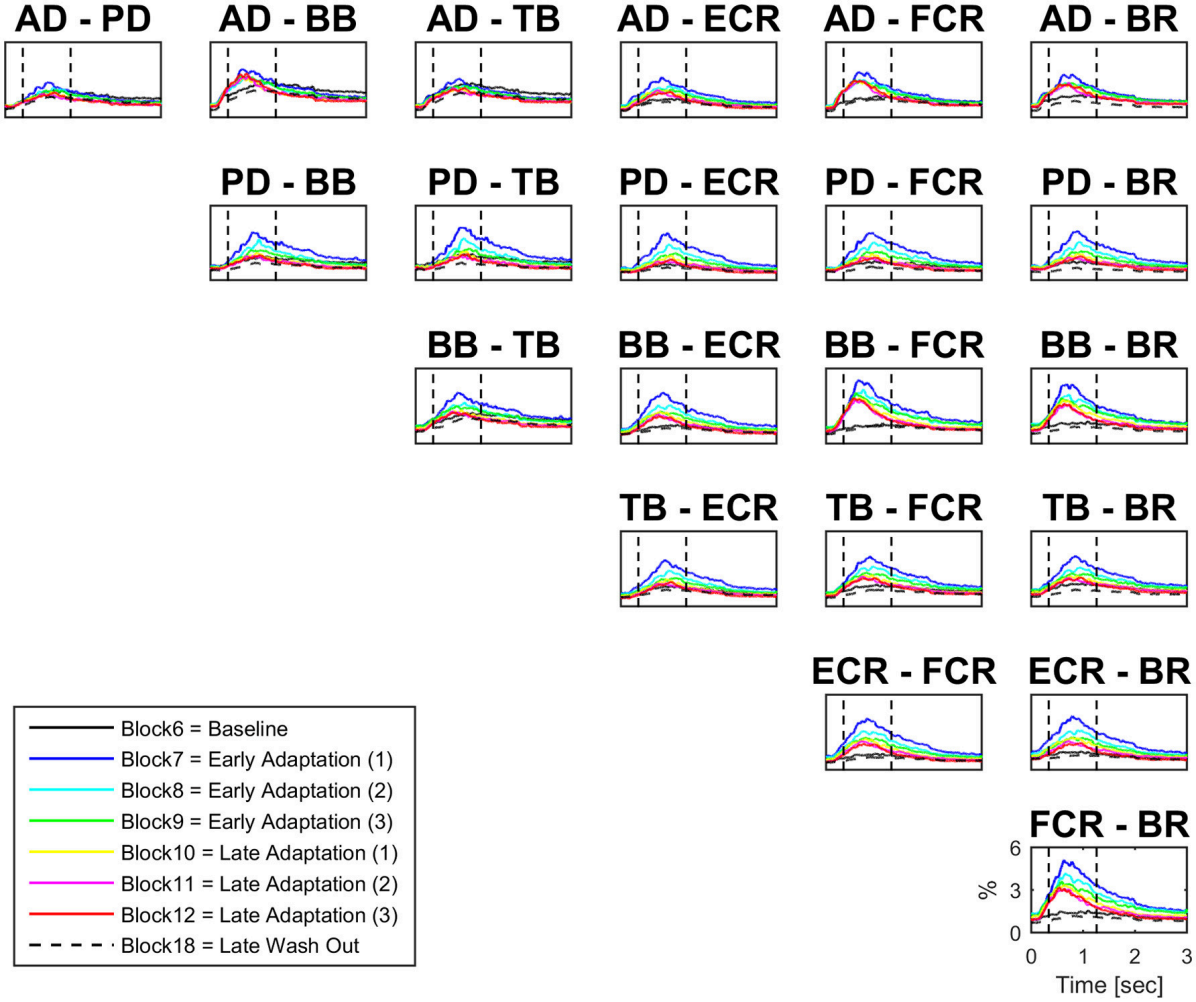
**Block-by-block co-contraction profiles for 21 muscles pairs**. For each muscle pair block-by-block average (*N* = 16) co-contraction profiles (from visual cue to 3 s afterwards) have been color coded to describe the evolution of co-contraction over the adaptation period. The most active muscles during adaptation are also the most co-active (e.g., BB-FCR).

There were significant increases in block-by-block peak co-contraction in the majority, but not all, muscle pairs (18 out of 21; Figure 3). There were no significant differences in peak co-contraction between baseline and wash-out blocks in any of the muscle pairs. The highest values of peak co-contraction were always in block 7 but only reached ~6% of maximal activation ratio for example in pairs BB-FCR and FCR-BR (Figure 3). There was also a statistically significant decrease in the peak co-contraction latency for AD-BB and BB-FCR during adaptation in comparison to baseline (both *p* < 0.001). On the other hand, PD-TB showed a prolongation of peak co-contraction latency in comparison to baseline during adaptation (*p* < 0.001).

### Intermuscular coherence

**Figure 4:**
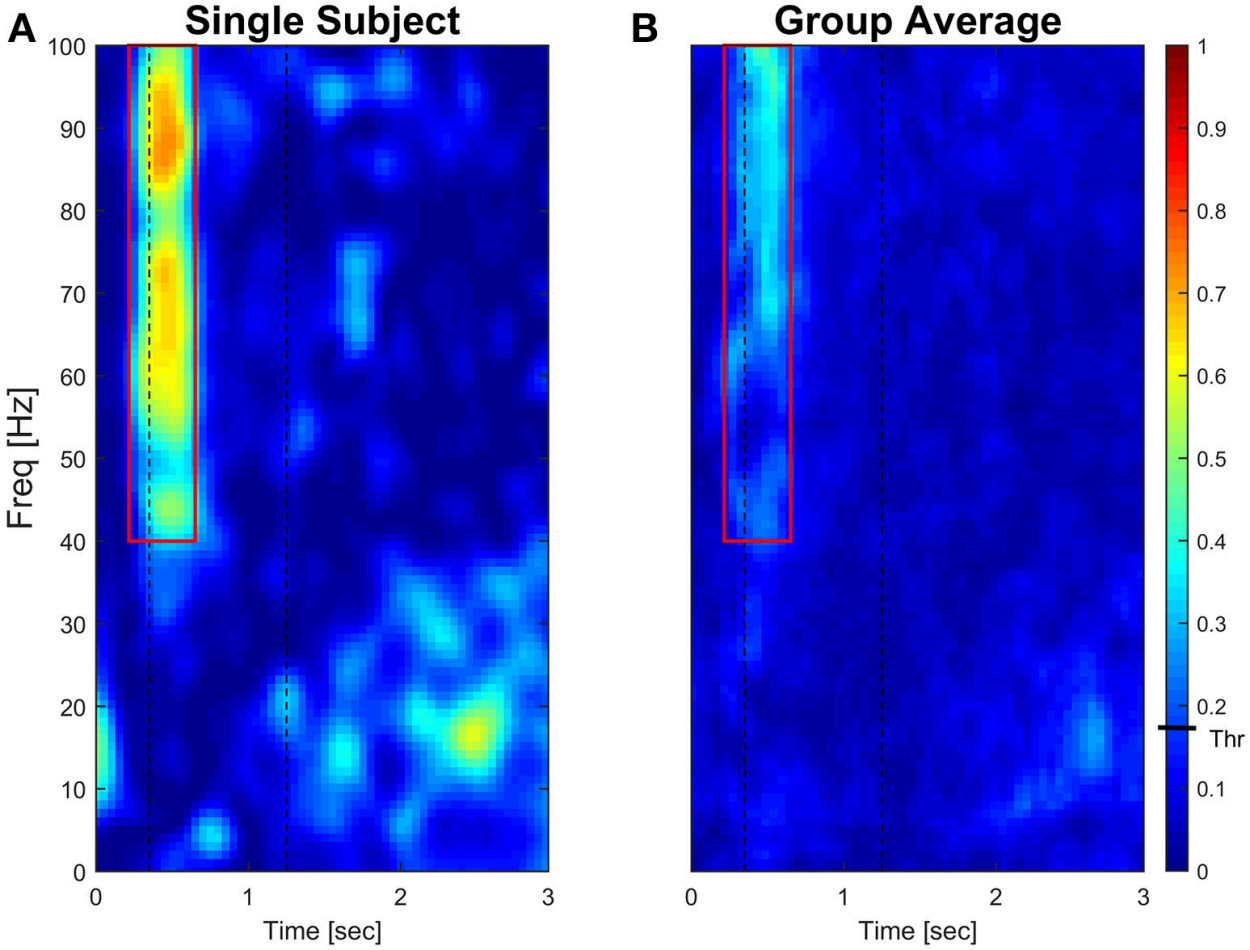
**Subject-specific and group-averaged time-frequency IMC during late adaptation (Block12) for PD-FCR**. Color-coded absolute IMC data from a typical subject **(A)** and averaged for the group (**B**; *N* = 16) during late adaptation (Block 12) for the PD-FCR muscle pair. A significant increase of coherence is shown in high-frequencies straddling the beginning of the movement both at subject and group level. Dashed black vertical lines represent averaged movement onset and offset for Block12. Continuous red lines represent window of interest straddling movement onset in the high-frequency range in which IMC absolute values are significantly different from zero. The confidence interval is represented by a black bar on color scale (Thr)–all colors above bar are significantly different from zero.

**Figure 5:**
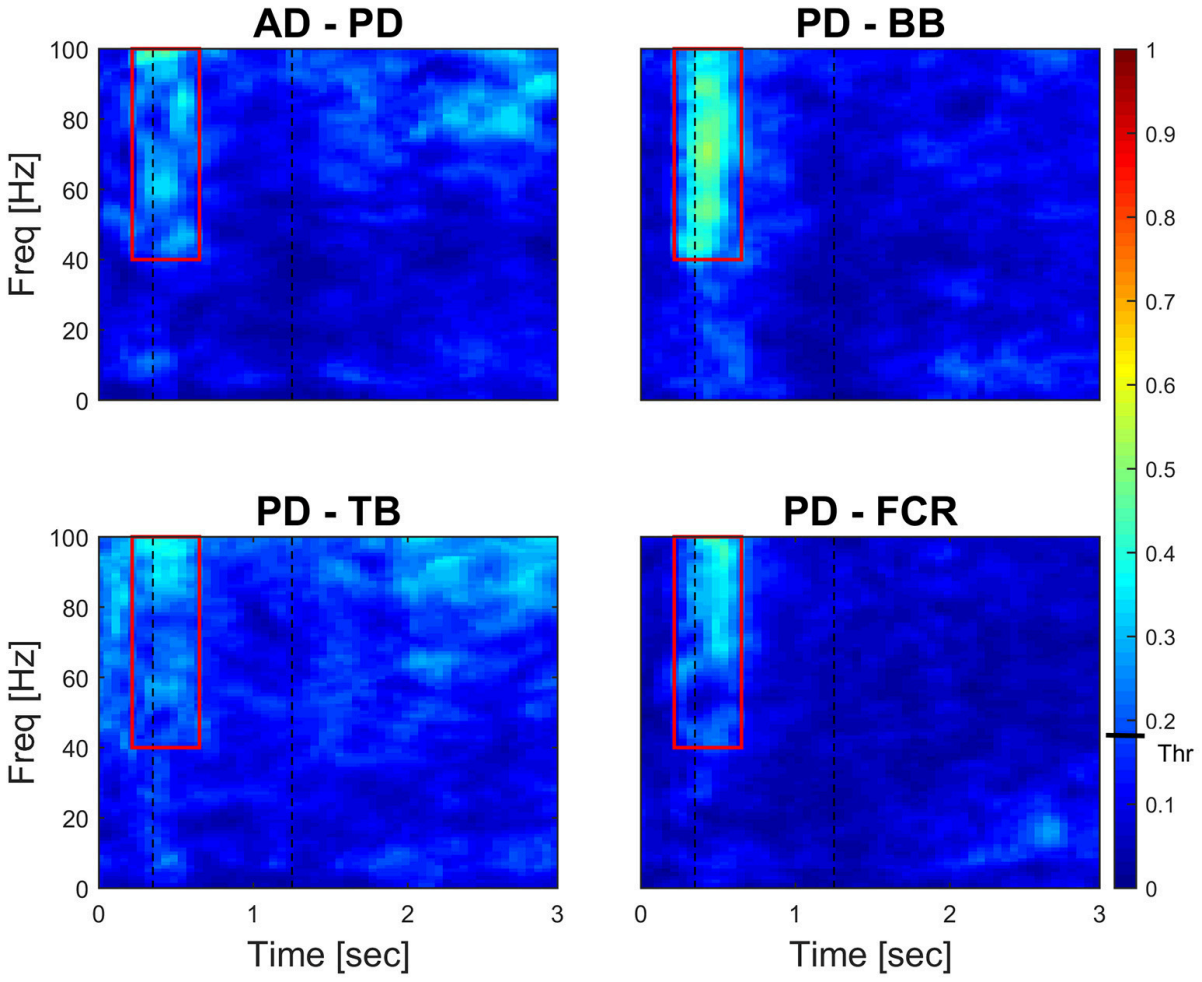
**Time-frequency IMC during late adaptation (Block 12)**. Color-coded averaged (*N* = 16) IMC during late adaptation (Block 12) for the four out of twenty-one possible muscles pairs of the right arm that demonstrated significant IMC. An increase of coherence is shown in high-frequencies straddling the beginning of the movement. Dashed black vertical lines represent averaged movement onset and offset for Block 12. Continuous red lines represent window of interest straddling movement onset in the high-frequency range in which IMC absolute values are significantly different from 0. The confidence interval is represented by a black bar on color scale (Thr)–all colors above bar are significantly different from zero.

Figure 4 (left panel) reports IMC in a typical subject with an area of interest straddling movement onset in the high-frequency range highlighted with red continuous lines. The increase of IMC in high-frequencies is also reflected at the group level, where values significantly different from 0 are visible in similar time-frequency areas of interest (Figure 4, right panel). Figure 5 illustrates absolute IMC values during late adaptation (block 12) for only the 4 (out of a possible 21) muscle pairs which demonstrated significant patterns of increased IMC over adaptation (Table 2).

**Table 2:** **Muscle pair co-contraction and coherence during motor adaptation**.

	**Muscle pair co-contraction and coherence during motor adaptation**								
	**Null field Block 6**	**Force field Block 7**	**Force field Block 8**	**Force field Block 9**	**Force field Block 10**	**Force field Block 11**	**Force field Block 12**	**Null field Block 18**	**Anova *p***
**CO-CONTRACTION PEAK (%)**									
AD-PD	4.43 (2.9)	5.34 (3.3)	4.48 (3.3)	4.08 (3.2)	3.86 (3.1)	4.04 (3.2)	3.66 (3.6)	3.11 (2.3)	NS
PD-BB	3.83 (2.3)	7.66 (5.3)^*^	5.866 (4.3)	4.56 (2.9)	4.09 (2.5)	4.23 (2.7)	3.77 (2.8)	2.53 (1.6)	0.001
PD-TB	4.34 (2.6)	9.69 (4.3)^*^	6.87 (2.9)^*^	5.44 (3.2)	4.94 (3.1)	4.93 (3.2)	4.11 (3.4)	2.63 (1.37)	0.001
PD-FCR	3.25 (2.4)	7.69 (3.8)^*^	5.89 (3.4)^*^	4.33 (2.9)	4.06 (2.6)	3.94 (2.9)	3.57 (3.1)	1.94 (1.2)	0.001
**CO-CONTRACTION PEAK LATENCY (ms)**									
AD-PD	495 (207)	600 (240)	619 (147)	587 (225)	544 (175)	446 (207)	425 (259)	554 (228)	NS
PD-BB	549 (162)	626 (176)	595 (163)	556 (163)	507 (176)	453 (155)	488 (218)	572 (254)	NS
PD-TB	399 (209)	638 (229)^*^	598 (114)^*^	621 (148)^*^	584 (154)^*^	605 (199)^*^	536 (186)	527 (220)	0.006
PD-FCR	458 (212)	603 (192)	642 (177)	562 (244)	549 (181)	517 (239)	471 (219)	480 (284)	NS
**IMC PEAK COHERENCE**									
AD-PD	0.40 (0.17)	0.47 (0.19)	0.51 (0.15)	0.52 (0.28)	0.57 (0.29)	0.58 (0.32)	0.63 (0.29)^*^	0.46 (0.23)	0.045
PD-BB	0.34 (0.16)	0.43 (0.16)	0.49 (0.24)	0.49 (0.23)	0.55 (0.25)^*^	0.65 (0.27)	0.61 (0.30)^*^	0.45 (0.12)	0.042
PD-TB	0.39 (0.24)	0.48 (0.19)	0.48 (0.15)	0.47 (0.06)	0.54 (0.22)	0.58 (0.22)	0.57 (0.25)	0.45 (0.22)	NS
PD-FCR	0.31 (0.23)	0.47 (0.24)	0.48 (0.28)	0.51 (0.28)	0.57 (0.28)^*^	0.56 (0.27)^*^	0.59 (0.29)^*^	0.31 (0.19)	0.007
**IMC PEAK LATENCY (ms)**									
AD-PD	200 (167)	117 (144)	133 (111)	197 (120)	144 (126)	106 (131)	147 (140)	136 (125)	NS
PD-BB	185 (157)	241 (156)	236 (147)	212 (124)	177 (136)	185 (174)	185 (134)	233 (183)	NS
PD-TB	165 (82)	155 (95)	145 (103)	168 (111)	144 (91)	141 (82)	126 (120)	145 (107)	NS
PD-FCR	191 (223)	161 (164)	166 (125)	139 (99)	165 (147)	112 (101)	179 (161)	248 (227)	NS
**IMC PEAK FREQUENCY (Hz)**									
AD-PD	79 (23)	76 (21)	82 (19)	79 (24)	72 (20)	74 (21)	71 (21)	80 (17)	NS
PD-BB	71 (22)	70 (22)	72 (22)	77 (21)	75 (18)	77 (18)	75 (15)	78 (18)	NS
PD-TB	81 (21)	75 (22)	76 (22)	77 (23)	77 (23)	84 (18)	80 (18)	77 (16)	NS
PD-FCR	64 (22)	76 (22)	78 (19)	80 (20)	83 (19)	86 (16)	85 (17)	65 (20)	0.008

Block-by-block mean values (SD) for muscle pair co-contraction (peak and peak latency relative to movement onset) and peak coherence, peak coherence frequency and latency (peak latency relative to movement onset). Repeated measures ANOVA p-values are reported in the last column on the right and statistically significant paired-samples t-test corrected for multiple comparisons (FDR) are highlighted with

* *(N = 16, Block 6 vs. Block_i_ with i = 7, …,12, 18; 7 comparisons)*.

#### Changes in co-contraction and IMC time-frequency mapping

**Figure 6:**
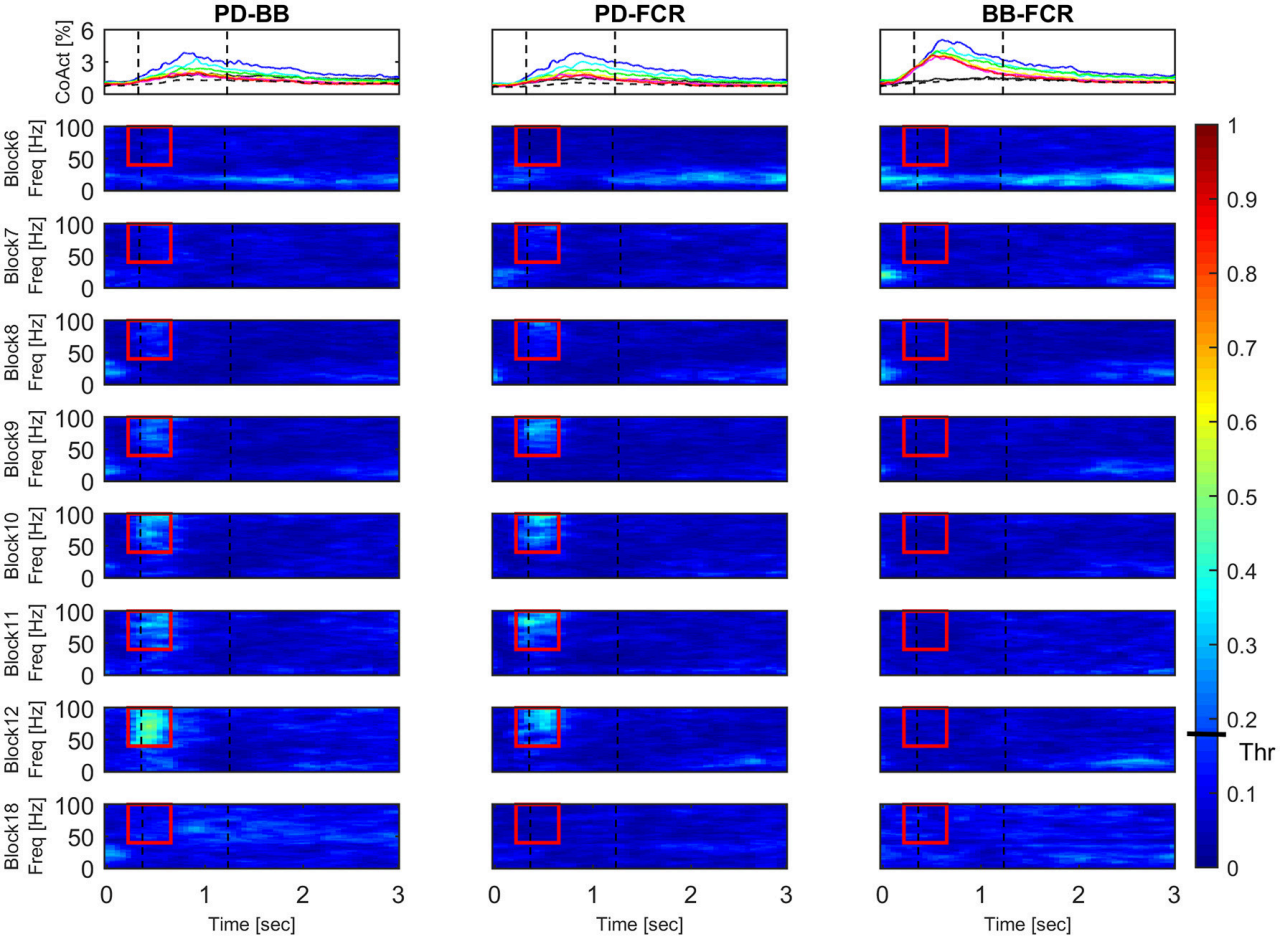
**Block-by-block evolution of co-contraction profiles (top panels) and intermuscular coherence statistic maps (lower panels) during motor adaptation for muscle pairs PD-BB, PD-FCR, and BB-FCR**. The co-activity of the three pairs of interest is described in terms of co-contraction and IMC across experimental blocks. Co-contraction averaged (*N* = 16) profiles (top panels) are color-coded from block 6 (baseline, black line), through all motor adaptation blocks (block 7, blue–block 12, red), to block 18 (late wash out, dashed black line). For a complete legend, see Figure 3. IMC is reported as absolute values in all blocks. Dashed black vertical lines represent averaged movement onset and movement offset in each block according to kinematic measures. This figure demonstrates that during motor adaptation there is a decrease of co-contraction during adaptation (Block 7–12) in some of the muscle pairs (PD-BB, PD-FCR) accompanied by an increase of high-frequency IMC. On the other hand, there is a constant increase in co-contraction during adaptation (Block 7–12, BB-FCR) with no significant IMC patterns. The confidence interval is represented by a black bar on color scale (Thr)–all colors above bar are significantly different from zero.

Figure 6 illustrates the block-by-block evolution of co-contraction profiles (top panels) and absolute IMC maps (i.e., using the IMC maps; lower panels) during motor adaptation for muscle pairs PD-BB, PD-FCR, and BB-FCR. Two phenomena appear in this representation: (1) an increase in IMC occurring at high frequencies (40–100 Hz) in a constrained time window, for example for the PD-FCR IMC, from ~250 to ~700 ms after the visual cue including movement onset at ~350 ms and (2) a significant reduction of IMC with respect to the baseline level (Block 6) in low frequencies (10–35 Hz) in some muscle pairs involving BB. These reductions in lower frequency IMC occurred before movement onset and after movement offset. They also occurred in frequencies commonly studied in isometric or quasi-isometric contractions, however this only occurred in a very few pixels in this study, therefore these have not been considered further.

Interestingly, co-contraction profile peaks decreased over adaptation blocks in muscle pairs PD-BB and PD-FCR but not BB-FCR, whereas high frequency IMC increased in PD-BB and PD-FCR but not in BB-FCR. For example in the PD-FCR muscle pair, there is a substantial increase in IMC between 40–100 Hz during later adaptation. A similar pattern emerges in the PD-BB and in the other two muscles pairs (AD-PD, PD-TB, Figure 4), although to a lesser degree. Within the reaching movement, all four IMC start to increase just after movement onset and terminate in mid-reach at ~700 ms and before movement offset.

#### High-frequency IMC

For each subject, the absolute peak IMC value was extracted in blocks 6, 7–12, and 18 from the time-frequency IMC map region-of-interest in the four representative muscles pairs that demonstrated a significant increase in high-frequency IMC. Frequencies and latencies of the absolute peak IMC were also statistically assessed further (see Table 2) in order to monitor any shift in the frequency and/or time domain.

Peak IMC values significantly increased across motor adaptation blocks and returned to baseline in complete washout in AD-PD, PD-BB, and PD-FCR (1 way repeated measures ANOVA *F* values all > 3.150; *p* values all < 0.045; Table 2), whereas there was a tendency toward significance for muscle pair PD-TB (*F* = 2.621, *p* = 0.052). Neither peak frequency (~70–80 Hz) nor peak IMC latency relative to movement onset (~350 ms) changed significantly across the 6 blocks of motor adaptation (1 way repeated measures ANOVA; *F* values all < 3.751; *p* values all > 0.05; Table 2).

## Discussion

### Novel findings

The present study describes the development of patterns of coherent muscle activity across a range of arm muscles during a force field motor adaptation paradigm. The findings are novel for this type of motor skill learning and the use of IMC in this study is an alternative to the traditional measures of integrated muscle activation or co-contraction/co-contraction. We demonstrated, for the first time, that there are muscle-pair specific, time- and frequency-dependent changes in intermuscular coupling during the dynamic non-isometric stage of a reaching task during motor adaptation. These findings build on changes in kinematics and basic muscle activity and co-contraction that others have documented for this specific type of motor adaptation (Milner and Franklin, 2005; Darainy and Ostry, 2008; Huang and Ahmed, 2014).

### Kinematic and basic muscle activity indices of motor adaptation

The typical disturbance of reaching kinematics by a velocity-dependent force field was accompanied by a more rapid and increased muscle activity as shown previously; both kinematics and peak EMG returned to their normal values once the effects of the perturbation were washed away (Thoroughman and Shadmehr, 1999). Motor adaptation represents a change in the capability of responding to specific tasks or external stimuli, given by practice or novel experience. In force-field learning protocols it usually manifests as rapid improvements in trajectory at the beginning of the session (first 20 trials or so) followed by a slower rate of improvement over the following trials (Franklin et al., 2003; Osu et al., 2003; Milner and Franklin, 2005; Smith and Shadmehr, 2005; Smith et al., 2006; Hunter et al., 2009). Our study was similar, such that complex changes in muscle activity enabled exponential reductions in summed error during adaptation (Shadmehr and Holcomb, 1997; Milner and Franklin, 2005; Huang and Ahmed, 2014). Specifically, muscle activities did increase significantly and remained highly elevated during adaptation in those muscles expected to counteract a clockwise force-field, which is Biceps Brachii and Flexor Carpi Radialis (Figure 2).

### Intermuscular coherence during motor adaptation

Time-frequency mapping demonstrated a progressive significant increase of IMC from early to later adaptation in a high frequency band in muscle pairs AD-PD, PD-BB, PD-TB, and PD-FCR, but not in lower frequency bands, despite the majority of muscle pairs demonstrating co-contraction in early adaptation (see Figure 5). The progressive increase in high-frequency IMC during the motor adaptation blocks occurred against a background of an elevated stable peak force (comparing Figures 1, 4) and thus was not solely related to force production *per se*.

The increase in IMC only appeared between certain muscle pairs (and not all) suggesting that it was not a result of systematic “cross-talk” between muscle activities and some muscles were anatomically distant and unlikely to exhibit cross-talk (e.g., PD-FCR muscle pair). Moreover, BSS using the JADE algorithm was also applied to minimize crosstalk (see Supplementary Figures 1, 2) and maximize the source content of each muscle signal (Cardoso and Souloumiac, 1993; Kilner et al., 2002). An effect of muscle fatigue on IMC (i.e., increase) was also unlikely as peak forces produced and peak EMG amplitudes during motor adaptation were small fractions of maximal values in this group of subjects (Danna-Dos Santos et al., 2010; Kattla and Lowery, 2010; Beck et al., 2014). Moreover, an additional investigation of EMG median frequency changes reported in Supplementary Material didn't show any effects of fatigue (see Supplementary Table 2). Finally, possible mechanical or physiological tremor associated with the reaching movement would be constrained to very low frequency bands (0–8 Hz) and thus unlikely to contribute to the high frequency IMC demonstrated in this study (He et al., 2015; van der Stouwe et al., 2015).

The most interesting aspect of high-frequency IMC is the progressive increase across blocks of force field adaptation whilst the kinematics indicative of motor adaptation (i.e., summed error) and muscle pair co-contraction decrease (see Figure 6). Furthermore, the increase in high-frequency IMC occurs during a time period after the visual cue typically used for measuring peak EMG and co-contraction in previous studies (e.g., Thoroughman and Shadmehr, 1999). IMC wanes away after this time period within a trial, so it appears not related to muscle activity during the holding position at the peripheral target (i.e., a quasi-isometric condition; Baker et al., 1999; Kilner et al., 1999).

High-frequency IMC has not received great attention (Grosse et al., 2002), but there is precedent for its occurrence between bilateral human respiratory muscles (Carr et al., 1994) and in eye movements (Brown and Day, 1997). A novel experimental study of IMC in lower limb muscles during pedaling (De Marchis et al., 2015), demonstrated significant gamma-band IMC between knee extensors whose function was to generate power and propel the crank during the pedaling task, suggesting a direct relationship between IMC, muscle coordination optimization and ultimately functional force production for the pedaling movement.

Recent studies further supported the hypothesis that multiple muscles involved in a complex task are coordinated through a neural synchronization strategy leading to the optimization of “intermuscular coupling” (Farmer, 1998; Charissou et al., 2016; de Vries et al., 2016). IMC might be representative of diverging pathways (both efferent and afferent) controlling multiple muscles coordination (Nazarpour et al., 2012), whereas corticomuscular coherence (CMC) might reflect direct descending pathways to individual muscles. High-frequency CMC becomes prominent during tasks involving strong (Mima et al., 1999) or dynamically modulated contractions (Brown et al., 1998; Omlor et al., 2007). Interestingly, CMC in alpha and gamma frequency bands appears also to be related to the reorganization of corticomuscular interactions during transitions between sensorimotor states (Mehrkanoon et al., 2014). In this specific study, gamma and alpha activities play a bilateral dual-band synchrony role presumably joining motor (descending, gamma) and sensory (ascending, alpha) processing during error corrections. Alpha frequency contribution here was however small, presumably due the constrained movement and dynamicity of the task.

We argue that our IMC findings reflect a neural strategy of multiple muscles coordination during a dynamic task in which subjects had to optimize their performance and minimize the movement error. As a matter of fact, the muscles demonstrating increased IMC during adaptation in our study included those most required during adaptation (i.e., BB and FCR peak amplitudes). In other words, coherent contributions may have originated between several “primary” and “associated” muscles (e.g., AD, PD, BB, TB, and FCR) in order to coordinate them and generate a functional force that could counteract the perturbation, whilst generating a propelling force strong enough to overcome the perturbation. Specifically, whereas AD and PD/TB are normally activated to break the reaching movement (i.e., *function-specific muscles*), BB is known to activate during adaptation to a clockwise force field when reaching in a 135° direction (Thoroughman and Shadmehr, 1999). Our study extended the number of muscles investigated and showed that also FCR is directly involved in counteracting the provided force field (Figure 2). Overall, this gives additional credence to the possibility of a functional relationship between IMC and intermuscular coupling optimization between muscles responsible for force production/resistance.

Importantly, according to a recent computational investigation based on a biologically-inspired mathematical model of the behavior of a typical motoneuron (MN) pool (Watanabe and Kohn, 2015), high frequency neural oscillations to muscles may recruit a larger number of motoneurons available from a specific muscle pool which leads to a greater or similar muscle force production but at a lower energetic cost. Moreover, stronger high-frequency synchronization at motor unit level during dynamic in comparison to isometric contractions has been recently shown and linked to a neural optimization strategy for the musculoskeletal system during complex tasks (Mohr et al., 2015). Although we did not measure single motor unit activation in this study, we believe that the reported increase of high–frequency IMC in later adaptation in our work is a plausible mechanism underpinning the gradation of optimal intermuscular coupling for a reduced or maintained level of co-contraction and for the previously observed gradual reduction of metabolic cost during the motor adaptation process (Huang and Ahmed, 2014).

The IMC approach reveals an added value with respect to other methodologies, e.g., co-contraction analysis. As Figure 3 clearly shows, all muscles exhibit co-contraction in the early stages of adaptation, i.e., in the early trials of force-field application. All of them however then experienced a reduction of co-contraction. Considering this common co-contraction phenomenon (between close and distant muscles) during early adaptation, IMC appears more valid in extracting the most relevant functional contribution as demonstrated elsewhere (De Marchis et al., 2015). From a neuromechanical point of view, reaching during motor adaptation is characterized by subjects aiming to improve and eventually stabilize their performance, which probably explains why a significant coherent contribution emerges (Omlor et al., 2007). Lastly, IMC has been demonstrated to be unaffected by age *per se*, consequently representing a strong candidate biomarker for neurological changes in muscle activation (Jaiser et al., 2016).

Since motoneuronal discharge is the last stage of the neural pathway in motor control, it is possible that both cortical and peripheral neuronal inputs are contributing to the high frequency synchrony. It has been previously suggested that IMC may accurately describe descending cortical drives as a form of cortico-muscular coupling (Brown et al., 1999; Grosse et al., 2002). However, IMC as used here is not able to disentangle the neural circuits responsible for synchrony and it may be the result of a combination of cortical, cerebellar (as demonstrated in a very similar experimental paradigm by Krebs et al., 1998) and reticulospinal circuits as well as local spinal/corticospinal sensorimotor feedback loops (Lemon, 2008). Of note, is that high frequency IMC in respiratory and eye muscles is considered to have significant inputs of brainstem origin (Grosse et al., 2002). Thus, the adaptation of IMC in the present study is likely a composite of several neural pathway outputs (Lemon, 2008). Because of the ambiguity of the underlying mechanisms contributing to IMC (Héroux and Gandevia, 2013), further studies should provide evidence of cortico-muscular coherence (CMC) in order to disentangle cortical from non-cortical contributions to neural drives to muscles during motor adaptation (Boonstra, 2013). Moreover, the motor adaptation process was not complete as summed error did not fully return to that of late familiarization, so it is not possible to measure how/if IMC would have developed further or how/if IMC would have decreased in parallel with the full decrease of error and of learning itself. It would be interesting to test whether changes in IMC become substantiated and robust in longer term motor adaptation protocols and follow the “savings” measured in follow-up adaptation sessions on future days/weeks (Haith et al., 2015; Huberdeau et al., 2015). Evidence of high frequency activity may give crucial physiological insight into mechanisms of functional recovery and, following the examples found in the literature (Nishimura et al., 2009; Kisiel-Sajewicz et al., 2011; Fisher et al., 2012; Bravo-Esteban et al., 2014), we suggest high frequency IMC (40–100 Hz) should be further investigated in long term neurological conditions such as stroke.

In conclusion, we propose that during motor adaptation, high-frequency IMC develops over time in order to optimize the motor output and the final goal, which is the reduction of the kinematic error and secondarily the energetic cost. Thus, in later adaptation, coherent contributions between several muscles (i.e., increased IMC between pairs of muscles) functionally promote performance improvement through an optimal intermuscular coupling whilst maintaining a constant peak force production at a reduced energetic cost (Watanabe and Kohn, 2015). Consequently, performance improves by reducing the trajectory path error not via a further increase of gross EMG activity or force *per se*, but rather by increasing the “orchestrated” involvement of several upper limb muscles.

## Author contributions

SP and DT: Study concept and design, data acquisition, data analysis and interpretation, statistical analysis, drafting/revising the manuscript for content. MD: Data acquisition, drafting/revising the manuscript for content. HA and UN: Drafting/revising the manuscript for content. All the authors revised the final version of the manuscript.

### Conflict of interest statement

The authors declare that the research was conducted in the absence of any commercial or financial relationships that could be construed as a potential conflict of interest.
