# Supplementary material for: High-Frequency Intermuscular Coherence between Arm Muscles during Robot-Mediated Motor Adaptation
Source: Front Physiol. 2017 Jan 9;7:668. doi: 10.3389/fphys.2016.00668 (PMC5220015; doi:10.3389/fphys.2016.00668)
Supplement: Supplementary file 1 [file DataSheet1.DOCX]

Supplementary Material

**High-Frequency Intermuscular Coherence between Arm Muscles during Robot-Mediated Motor Adaptation.**

**Sara Pizzamiglio, Martina De Lillo, Usman Naeem, Hassan Abdalla, Duncan L. Turner^*^**

***Correspondence:**Prof. Duncan L. Turner
[d.l.turner@uel.ac.uk](mailto:d.l.turner@uel.ac.uk)

# Supplementary Figures and Tables

## Supplementary Figures


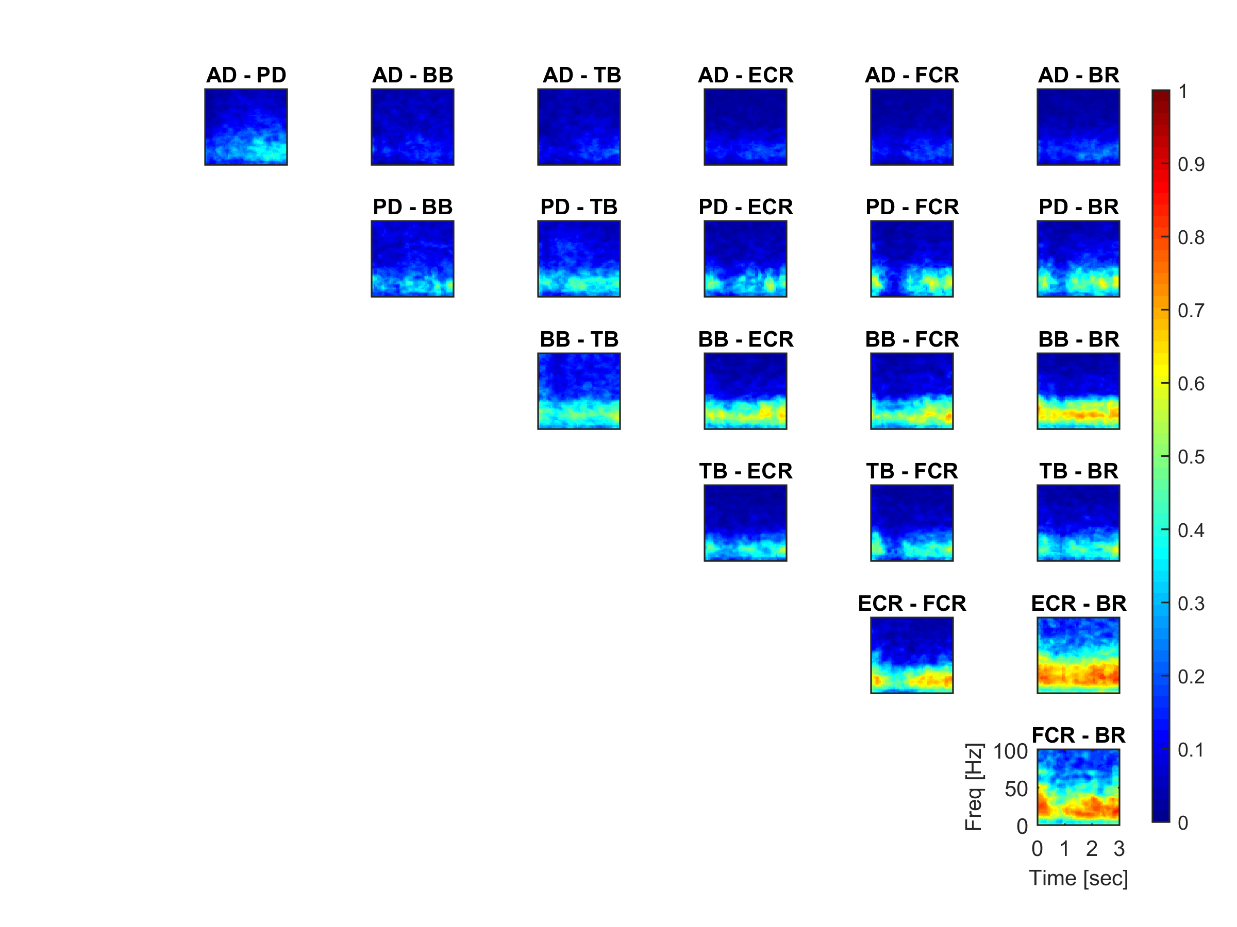


Supplementary Figure 1 – IMC during Block6 (end of Familiarization condition) without Blind Source Separation. Unrealistic values of coherence (1) are visible in muscles pairs including anatomically close muscles (e.g. forearm muscles).


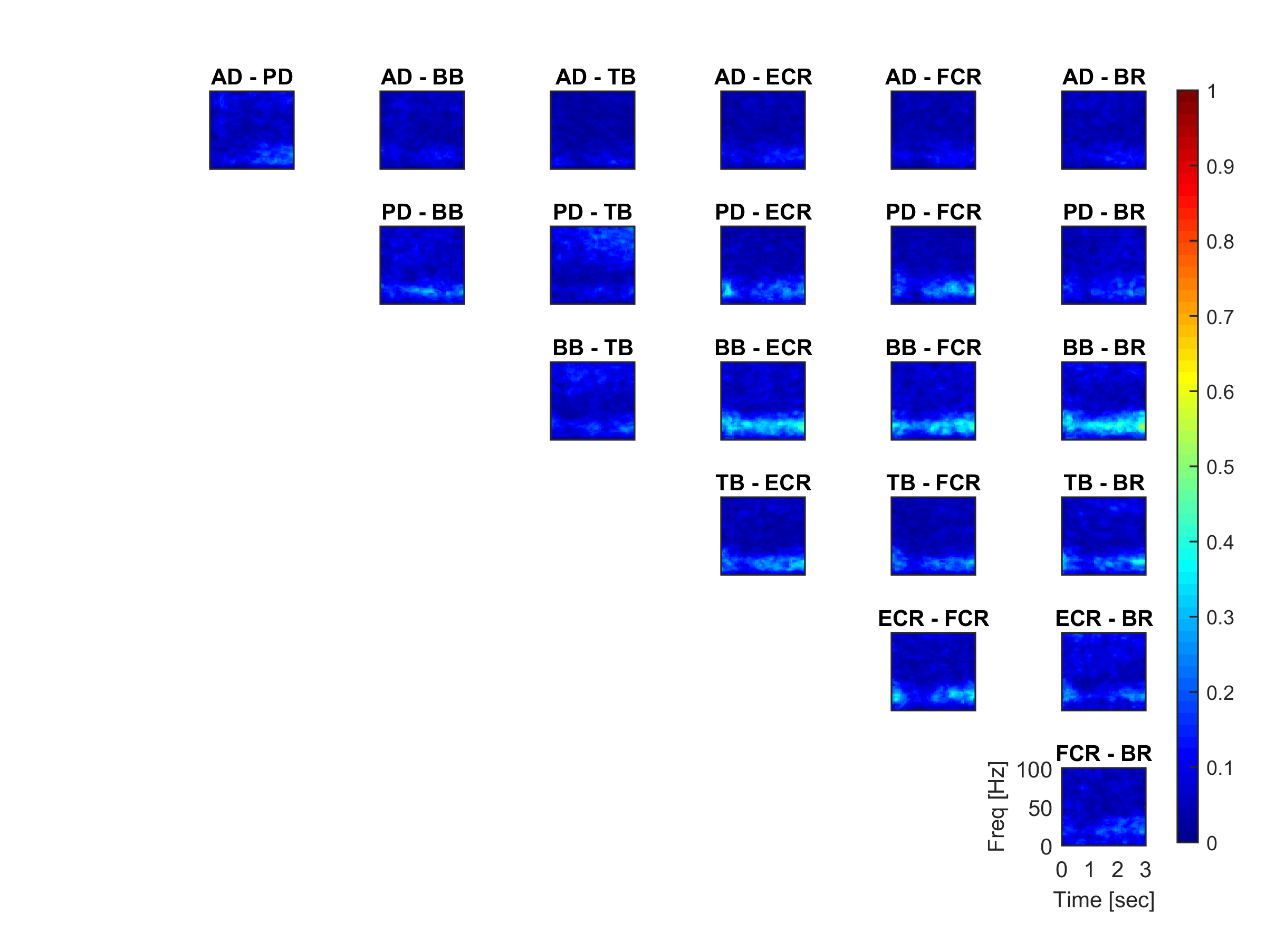


Supplementary Figure 2 - IMC during Block6 (end of Familiarization condition) with Blind Source Separation. Realistic values of coherence are visible in all muscles pairs including also those between anatomically close muscles (e.g. forearm muscles).


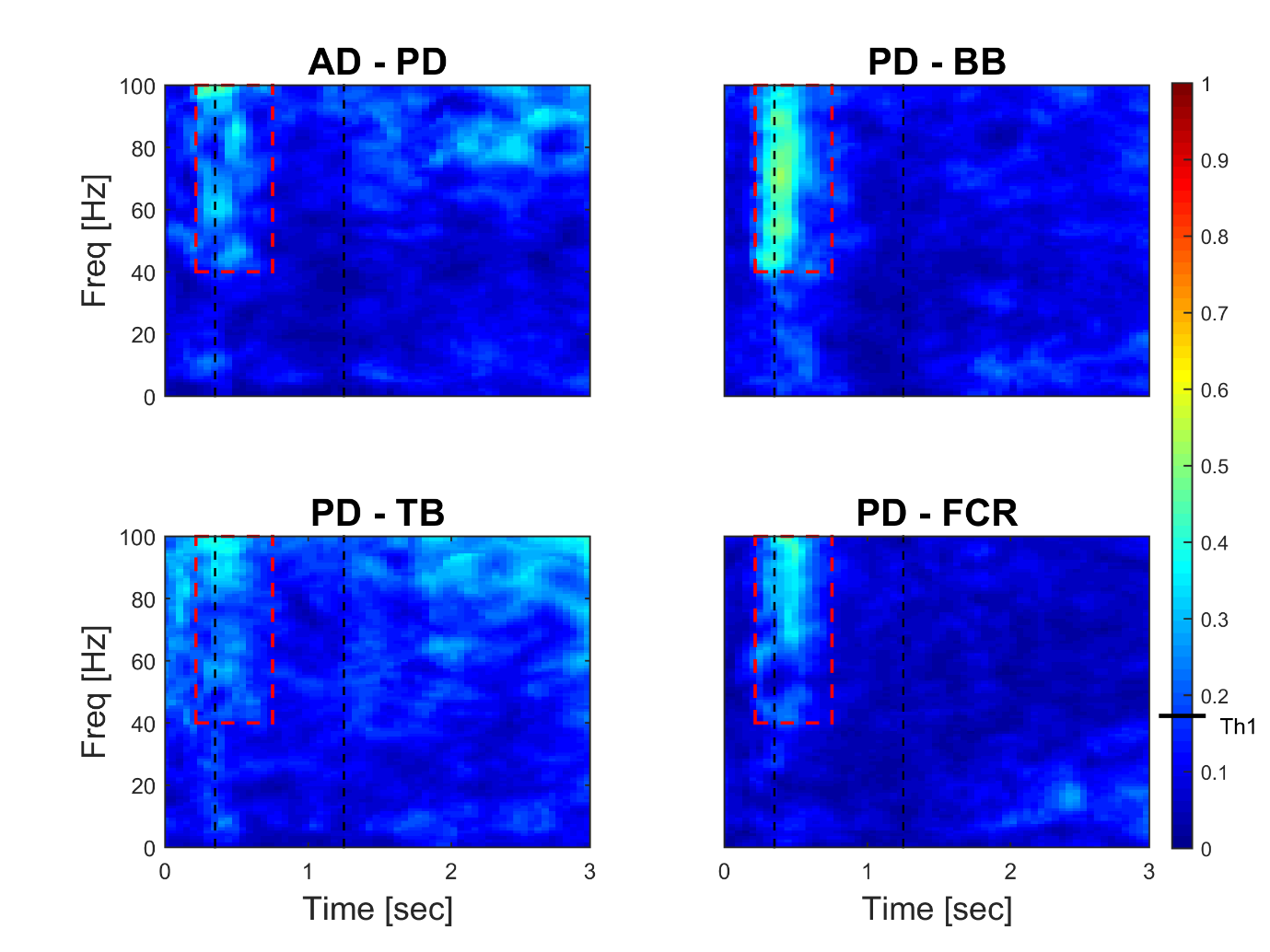


Supplementary Figure 3 – Group IMC and respective Confidence Interval threshold (Th1 = 0.1810) at 95% level of significance (α = 0.05). The area of significant difference of IMC from 0 of interest for the study is highlighted in red dashed lines.


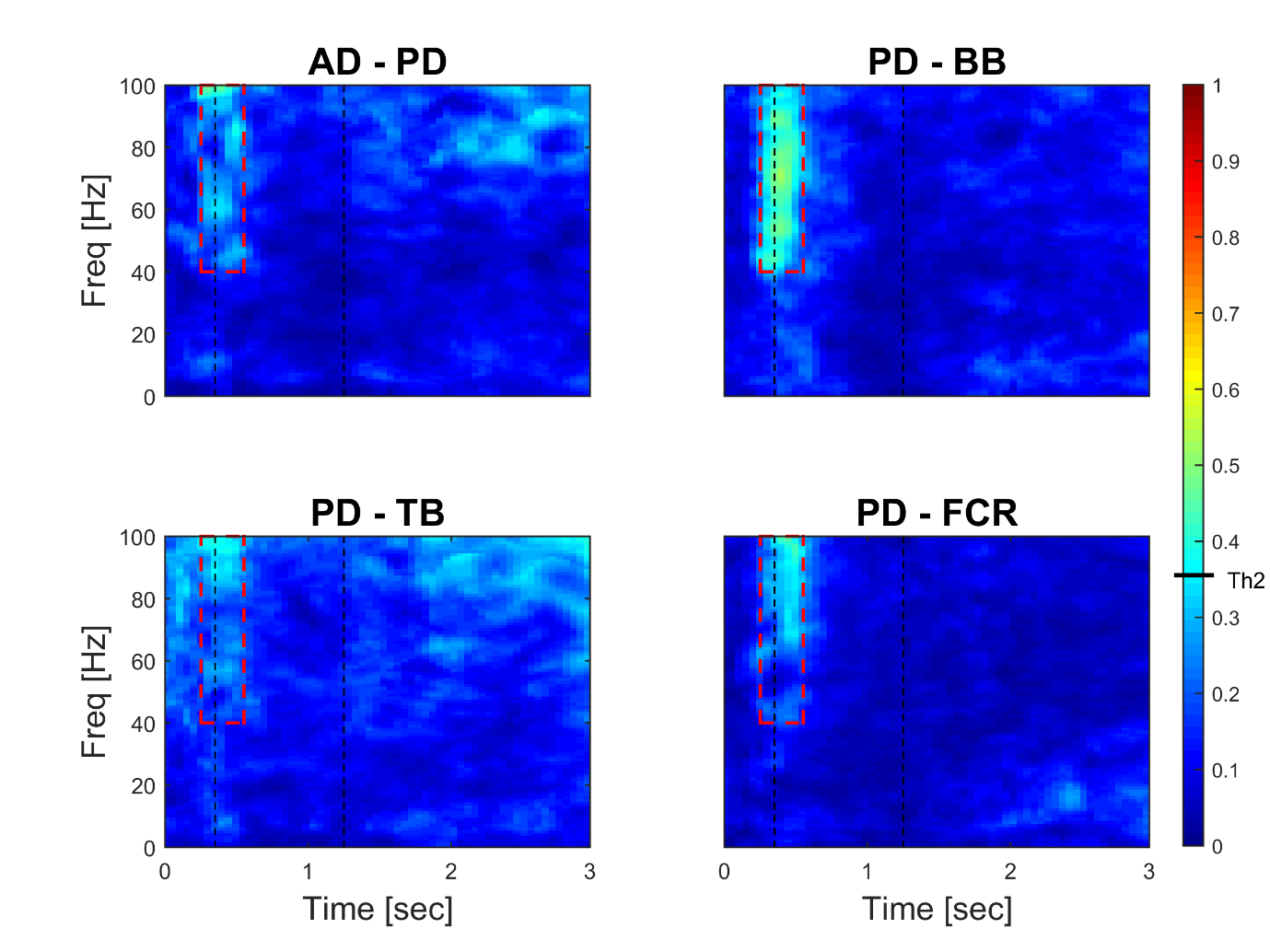


Supplementary Figure 4 – Group IMC and respective Confidence Interval threshold (Th2 = 0.3690) at 99.9% level of significance (α = 0.001). The area of significant difference of IMC from 0 of interest for the study is highlighted in red dashed lines. The area is smaller than in Supplementary Figure 3.


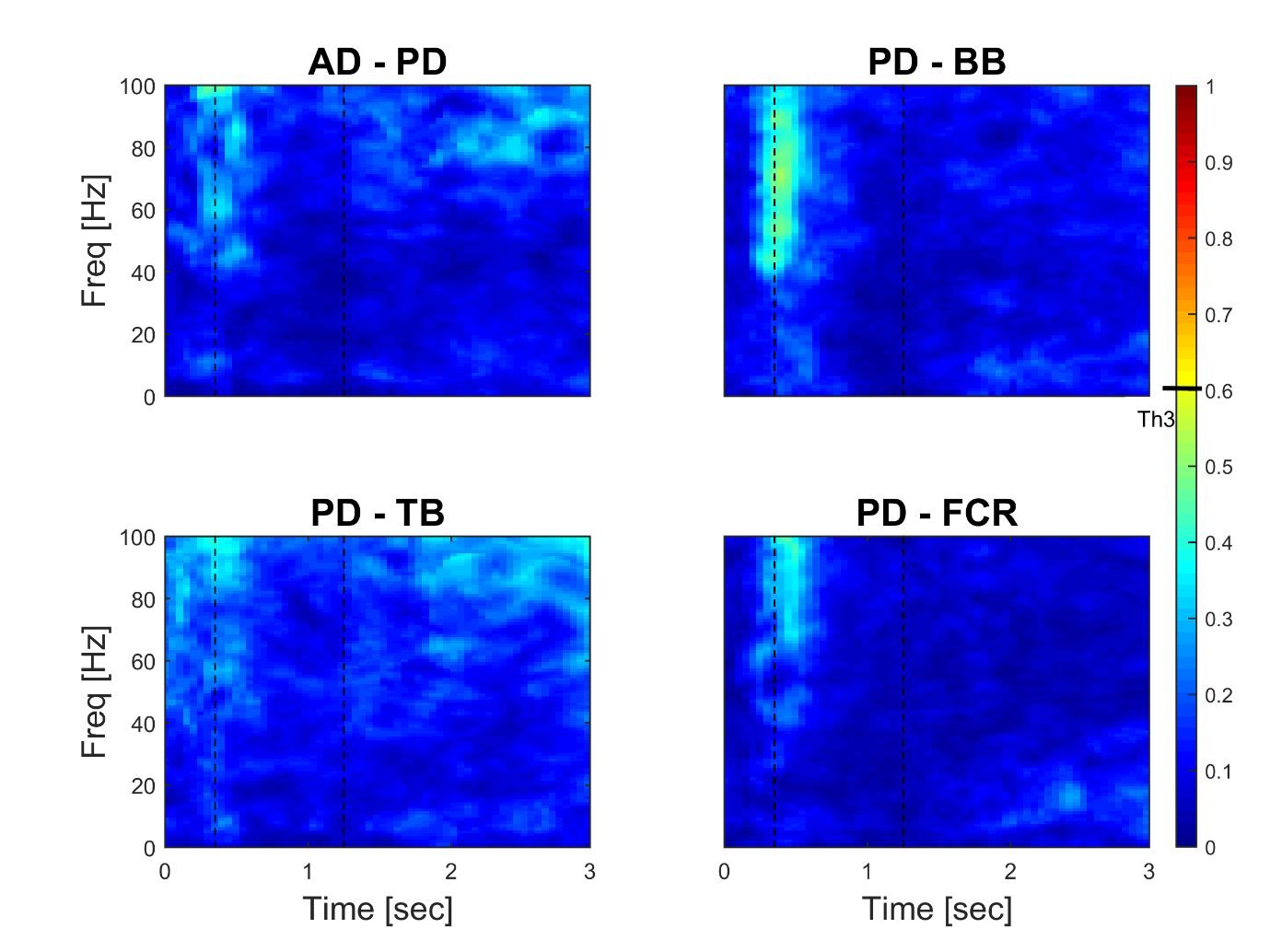


Supplementary Figure 5 - Group IMC and respective Confidence Interval threshold (Th3 = 0.6060) according to Sidak correction for multiple comparison. The threshold reached an unrealistic value due to the high number of comparisons considered (100x60 = 60000 pixels).

**
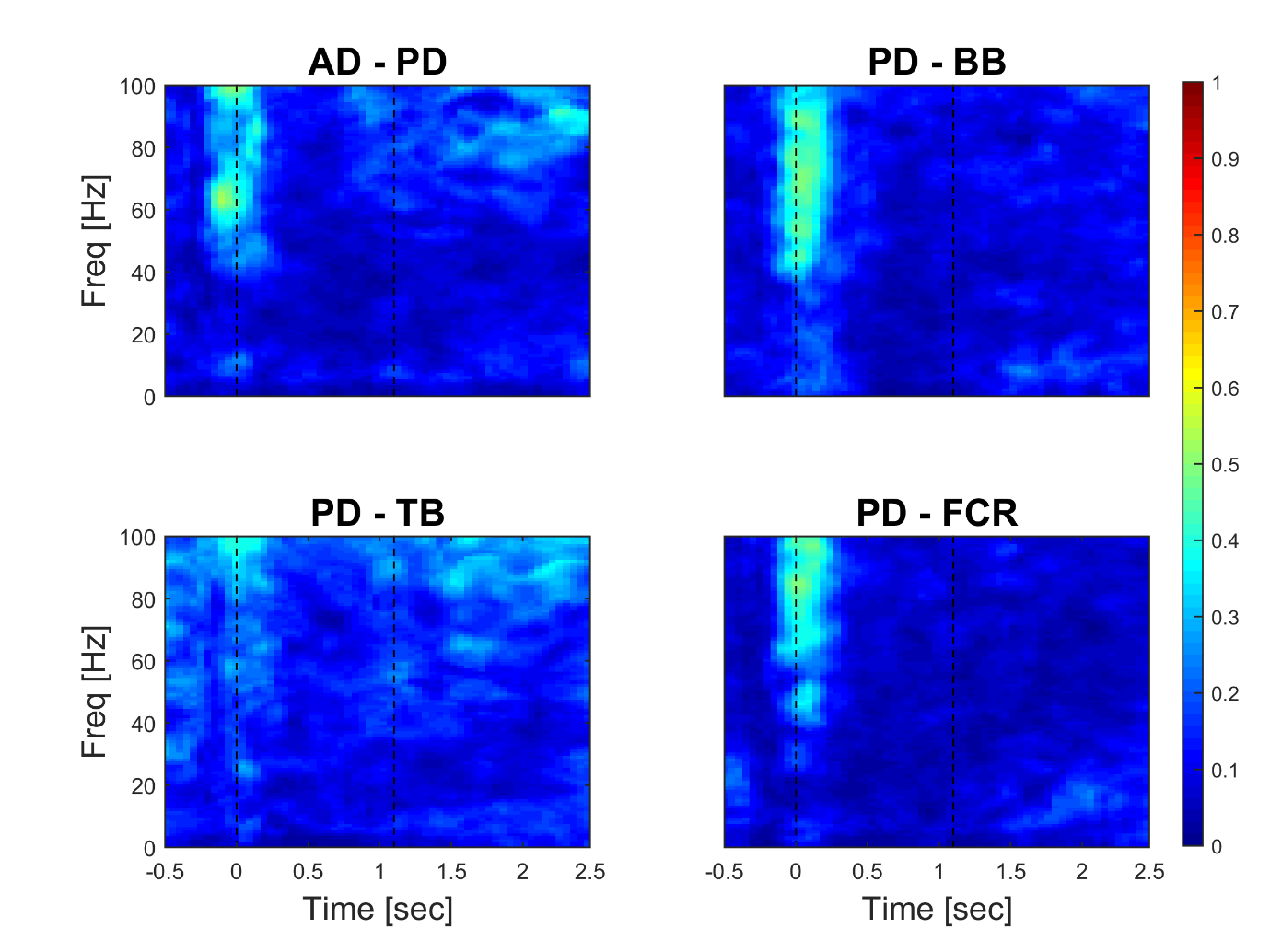
**

Supplementary Figure 6 - Group IMC for the 4 representative muscle pairs in Block12 with trials defined from -500 ms before to 2500 ms after Movement Onset (t = 0). Increase in high-frequency IMC is still evident and straddling movement onset.

**Supplementary Table 1** - Correlation results. Data from individual subjects were correlated resulting in a correlation coefficient for each subject. The distribution of correlation coefficients were tested against zero using a one-sample t-test. * Significance. α = 0.005.

| **Correlations** | | | |
| --- | --- | --- | --- |
|  | Coh vs. CoAct | Coh vs. SummErr | CoAct vs. SumErr |
| AD-PD | p = 0.057 | p = 0.207 | p = 0.071 |
| PD-BB | p = 0.276 | p = 0.213 | p = 0.007* |
| PD-TB | p = 0.108 | p = 0.036* | p < 0.001* |
| PD-FCR | p = 0.650 | p = 0.368 | p = 0.004 * |

A second type of correlation analysis had been also carried out following these steps:

- Evaluation of difference between Block12 (late adaptation) and Block7 (early adaptation) of peakCoh and peakCoAct in representative muscles pairs and of Summed Error for each subject;
- Correlation between measures from i = 1:16 subjects.

No significance was observed in the following correlations:

- PD-BB, diff_peakCoh vs. diff_SumErr: r = 0.4, NS
- PD-TB, diff_peakCoh vs. diff_peakCoAct: r = -0.4767, NS
- PD-FCR, diff_peakCoh vs. diff_peakCoAct: r = -0.4788, NS

Supplementary Table 2 - EMG Median Frequency summary. Friedman p-values are reported in the last column on the right, and statistically significant Wilcoxon test corrected for multiple comparisons (FDR) are highlighted with * (N = 16). Comparisons are between Block6 vs. Block_i_ with i = 7,…,12, 18.

| **Muscles Median Frequency** | | | | | | | | | |
| --- | --- | --- | --- | --- | --- | --- | --- | --- | --- |
|  | Null Field | Force Field | Force Field | Force Field | Force Field | Force Field | Force Field | Null Field | Anova |
|  | Block6 | Block7 | Block8 | Block9 | Block10 | Block11 | Block12 | Block18 | p |
| Median Freq [Hz] |  | | | | | | | | |
| AD | 77 (29) | 71 (24) | 78 (24) | 73 (23) | 72 (18) | 76 (15) | 65 (17) | 79 (25) | N.S. |
| PD | 76 (30) | 54 (12) * | 59 (22) * | 58 (21) * | 62 (20) * | 61 (21) * | 65 (20) | 74 (21) | 0.008 |
| BB | 107 (63) | 75 (30) | 72 (35) | 76 (28) | 72 (25) | 73 (22) | 70 (24) | 81 (38) | N.S. |
| TB | 97 (64) | 79 (40) | 81 (34) | 76 (37) | 86 (35) | 84 (29) | 87 (40) | 81 (34) | N.S. |
| ECR | 113 (35) | 94 (29) | 100 (38) | 106 (41) | 113 (38) | 113 (43) | 112 (44) | 117 (38) | N.S. |
| FCR | 99 (43) | 83 (23) | 81 (30) | 80 (18) | 77 (18) | 84 (19) | 81 (27) | 79 (34) | N.S. |
| BR | 115 (46) | 82 (29) | 89 (24) | 90 (37) | 96 (34) | 94 (27) | 94 (25) | 97 (40) | N.S. |

Supplementary Table 3 - Block-by-block mean values (SD) for muscle peak coherence, peak coherence frequency and latency evaluated in trials defined around Movement Onset (i.e. latency values could also be negative, alias before movement onset). Repeated measures ANOVA p-values are reported in the last column on the right and statistically significant paired-samples t-test corrected for multiple comparisons (FDR) are highlighted with * (N = 16, Block 6 vs. Block_i_ with i = 7, …, 18; 7 comparisons).

| **Muscle pairs coherence during motor adaptation** | | | | | | | | | |
| --- | --- | --- | --- | --- | --- | --- | --- | --- | --- |
|  | Null Field | Force Field | Force Field | Force Field | Force Field | Force Field | Force Field | Null Field | Anova |
|  | Block6 | Block7 | Block8 | Block9 | Block10 | Block11 | Block12 | Block18 | p |
| Peak IMC |  | | | | | | | | |
| AD-PD | 0.40 (0.20) | 0.49 (0.21) | 0.51 (0.27) | 0.52 (0.28) | 0.56 (0.30) | 0.58 (0.33) | 0.62 (0.30) | 0.46 (0.25) | 0.061 |
| PD-BB | 0.32 (0.17) | 0.42 (0.17) | 0.47 (0.26) * | 0.50 (0.24) * | 0.54 (0.26) * | 0.53 (0.27) * | 0.60 (0.30) * | 0.43 (0.22) | **0.031** |
| PD-TB | 0.41 (0.24) | 0.47 (0.19) | 0.50 (0.14) | 0.49 (0.15) | 0.53 (0.22) | 0.58 (0.24) | 0.57 (0.24) | 0.44 (0.21) | 0.075 |
| PD-FCR | 0.30 (0.23) | 0.46 (0.25) | 0.48 (0.28) | 0.52 (0.29) * | 0.58 (0.28) * | 0.58 (0.28) * | 0.59 (0.28) * | 0.32 (0.18) | **0.008** |
| Peak IMC Latency |  | | | | | | | | |
| AD-PD | 97 (179) | 87 (193) | 81 (170) | 40 (150) | 28 (172) | 3 (174) | 37 (174) | 50 (171) | N.S. |
| PD-BB | 91 (191) | 162 (162) | 175 (173) | 112 (150) | 112 (175) | 44 (141) | 75 (169) | 109 (206) | N.S. |
| PD-TB | 69 (197) | 41 (183) | 78 (168) | 97 (177) | 66 (155) | 12 (123) | 91 (154) | 44 (171) | N.S. |
| PD-FCR | 19 (173) | 131 (178) | 94 (156) | 81 (140) | 131 (157) | 41 (113) | 94 (121) | 131 (211) | N.S. |
| Peak IMC Freq. |  | | | | | | | | |
| AD-PD | 78 (23) | 76 (19) | 87 (18) | 80 (21) | 69 (20) | 74 (19) | 69 (20) | 80 (16) | 0.006 |
| PD-BB | 74 (22) | 67 (20) | 72 (21) | 74 (22) | 78 (16) | 75 (18) | 76 (16) | 76 (22) | N.S. |
| PD-TB | 80 (23) | 79 (22) | 76 (22) | 80 (21) | 74 (22) | 83 (18) | 83 (20) | 77 (17) | N.S. |
| PD-FCR | 69 (24) | 79 (21) | 76 (19) | 80 (19) | 86 (17) | 86 (15) | 85 (17) | 67 (19) | 0.004 |
